# Metabolomic Evaluation of Chronic Periodontal Disease in Older Adults

**DOI:** 10.1155/2021/1796204

**Published:** 2021-11-18

**Authors:** Wellington F. Rodrigues, Camila B. Miguel, Ferdinando Agostinho, Gabriela V. da Silva, Javier E. Lazo-Chica, Sandra M. Naressi Scapin, Marcelo H. Napimoga, Carlos A. Trindade-da-Silva, José E. Krieger, Alexandre da Costa Pereira, Carlo J. Freire Oliveira, Siomar de Castro Soares, Carlos Ueira-Vieira

**Affiliations:** ^1^Postgraduate Course in Health Sciences, Federal University of Triângulo Mineiro, 38015-050 Uberaba, MG, Brazil; ^2^Institute of Genetics and Biochemistry, Federal University of Uberlândia, 38400-902 Uberlândia, MG, Brazil; ^3^Medicine Course, University Center of Mineiros-Unifimes, 75830-000 Mineiros, GO, Brazil; ^4^Postgraduate Course in Physiological Sciences, Federal University of Triângulo Mineiro, 38015-050 Uberaba, MG, Brazil; ^5^Heart Institute-INCOR, Faculty of Medicine, University of São Paulo, 05403-900 São Paulo, SP, Brazil; ^6^Cell Biology Laboratory, Institute of Biological and Natural Sciences of the Federal University of Triângulo Mineiro, 38015-050 Uberaba, MG, Brazil; ^7^Instituto Nacional de Metrologia, Qualidade e Tecnologia, 25250-020 Duque de Caxias, RJ, Brazil; ^8^Institute and Research Center São Leopoldo Mandic, São Leopoldo Mandic Faculty-SLMANDIC, 13045-755 Campinas, SP, Brazil

## Abstract

Periodontal disease is an infectious inflammatory disease related to the destruction of supporting tissues of the teeth, leading to a functional loss of the teeth. Inflammatory molecules present in the exudate are catalyzed and form different metabolites that can be identified and quantified. Thus, we evaluated the inflammatory exudate present in crevicular fluid to identify metabolic biological markers for diagnosing chronic periodontal disease in older adults. Research participants were selected from long-term institutions in Brazil. Participants were individuals aged 65 years or older, healthy, or with chronic periodontal disease. Gas chromatography/mass spectrometry was used to evaluate potential biomarkers in 120 crevicular fluid samples. We identified 969 metabolites in the individuals. Of these, 15 metabolites showed a variable importance with projection score > 1 and were associated with periodontal disease. Further analysis showed that among the 15 metabolites, two (5-aminovaleric acid and serine, 3TMS derivative) were found at higher concentrations in the crevicular fluid, indicating their potential diagnostic power for periodontal disease in older adults. Our findings indicated that some metabolites are present at high concentrations in the crevicular fluid in older adults with periodontal disease and can be used as biomarkers of periodontal disease.

## 1. Introduction

Molecules present in fluids in the oral cavity may indicate a relationship between processes linked to health and disease, as well as repair processes. Inflammatory proteins play different and important roles in oral cavity homeostasis including periodontal disease. Periodontitis is an infectious inflammatory disease that causes destruction of the tissues supporting the teeth. It significantly affects oral health and is the most common cause of tooth loss. Additionally, preclinical and clinical studies have demonstrated clear associations between periodontitis and various other conditions as well as systemic diseases.[1–6].

The relationship between the microbiota and immune system is critical to the maintenance of periodontal health; therefore, certain groups, such as older adults, are more susceptible to the development of chronic periodontal disease [1]. Events related to biological senescence predispose older adults to infections and conditions that increase morbidity and mortality [7]. This increased susceptibility in older adults is associated with a decrease in the normal functioning of the immune system. The responsiveness of the T lymphocyte population decreases with advancing age, resulting in reduced efficiency of monocytes and macrophages in destroying invading pathogens [8, 9], thereby allowing for the development of periodontal diseases.

Recent advances in metabolic studies have enabled the identification and quantification of different metabolites in normal individuals and those with certain diseases using metabolomics [10–12]. Metabolites are chemically diverse and can be classified into ionic species, alcohols, hydrophilic carbohydrates, volatile ketones, lipids, and organic acids [13]. In disease diagnosis, several efforts have been made to identify metabolites in the saliva of individuals with diseases that would provide new biological markers to aid the diagnosis of periodontal disease [14]. However, the metabolic profile during the senescence period of individuals who have periodontal disease has not been defined.

Given the heterogeneous characteristics of the diverse immune responses to infection, efforts have been made to identify markers in the fluids in the oral cavity, such as saliva and crevicular fluid, to predict the presence of periodontal disease and its stage [11, 15]. Because of differences in the immune systems of different age groups, including during the senescence period, our study is aimed at identifying metabolic biological markers for diagnosing chronic periodontal disease in older adults.

## 2. Materials and Methods

### 2.1. Inclusion and Exclusion Criteria

Individuals aged ≥65 years, nonsmokers, and with or without chronic periodontal disease were included in the study. Individuals diagnosed with diabetes mellitus (controlled or not), arterial hypertension, without at least three absorbent cones collected, edentate individuals, those undergoing antibiotic therapy, or who had recently been treated by odontology intervention were excluded from the study.

### 2.2. Collection of Biological Materials

Initially, a minimum effect size of 50% was considered between the samples obtained from individuals with and without periodontal disease (independent-samples *t*-test) for the sampling calculation. With a power of 80% (a priori power analysis), it was possible to estimate a sample number of 102 participants. The percentage of possible losses was considered 18% due to nonadherence or dropout, leading to a total of 120 research participants (*α* = 5%). Therefore, individuals in the study were grouped into those with chronic periodontal disease (periodontitis group; *n* = 60) and those without (healthy group; *n* = 60). The mean age of the study subjects was 70 years (65–80 years). Diseased individuals were enrolled following clinical evaluations and crevicular fluid sampling using absorbent cones at a probing depth of ≥5 mm.

Selection of study subjects: all the teeth in the mouth were probed. The periodontal disease outcome measures included clinical attachment loss and periodontal pocket depth. The periodontal pocket was defined as the measurement starting at 5 mm from the gingival margin to the bottom of the pocket. The gingival margin was measured from the cementoenamel junction to the gingival margin. The examiners measured probing depth and gingival margin at six sites per tooth for each fully erupted tooth, except the third molars, in each patient. Two skilled examiners were calibrated for periodontal assessments, so that the measurements were comparable. The clinical attachment levels were calculated, a periodontal diagnosis was provided, and the patients were classified into two groups after periodontitis assessment: healthy and periodontitis [16].

After identifying the participants, three cones were obtained from the different sites of each patient and transferred into tubes containing 500 *μ*L of protease inhibitor (complete; Roche, Basel, Switzerland). Immediately after collection, the tubes were submerged in liquid nitrogen and stored at -80°C. The volume used in each analytical run was determined after quantifying total proteins by the Bradford method [17].

### 2.3. Ethical Statements

All procedures were approved by the research ethics committee of the Federal University of Triangulo Mineiro (number: 017430/2014), registered in Plataforma Brazil, and followed National Health Council Resolution 466/2012. All participants provided a formal written consent to participate in the study.

### 2.4. Sample Processing for Metabolomics

The crevicular fluid samples were kept on ice until they were completely thawed, and a maximum of 100 *μ*L of fluid (depending on the volume of proteins in each sample) was added to a tube containing 300 *μ*L of metabolite extraction buffer (ice cold) containing acetonitrile, isopropanol, and ultrapure water (3 : 3 : 2). The mixture was then centrifuged at 15,800 × *g* at 0°C for 15 min to precipitating the proteins, and 350 *μ*L of the supernatant was transferred to a new tube. As an internal standard, 5 *μ*L of myristic acid (#366889; Sigma-Aldrich, St. Louis, MO, USA) was added at a concentration of 3 mg/mL. The metabolites were then dried in a SpeedVac (Thermo Fisher, Waltham, MA, USA) for 18 h and stored in a desiccator at 4°C until analysis.

The samples were subjected to shunt processes. First, 3 *μ*L of fatty acid methyl ester was added to control the retention time alignment during sample processing. Subsequently, 30 *μ*L of a solution of 40 mg/mL methoxyamine (#226904; Sigma-Aldrich) diluted in pyridine (#270407; Sigma-Aldrich) was added, and the pellet containing metabolites was homogenized and incubated for 16 h at 25°C under agitation at 650 rpm. After methoxyamination, 90 *μ*L of *N*-methyl-*N*-(trimethylsilyl)trifluoroacetamide (#69479; Sigma-Aldrich) with 1% trimethylchlorosilane (#89595; Sigma-Aldrich) was added, and the mixture was incubated for 90 min at 25°C under agitation at 650 rpm. The metabolites were centrifuged at 15,800 × *g* for 5 min at 23°C, and 100 *μ*L of the supernatant was transferred to a 2 mL amber vial. The samples were analyzed within 24 h of shunting.

### 2.5. Metabolomic Analyses

Samples were analyzed in triplicate using a gas chromatography/mass spectrometry (GC/MS) system (7890B GC/5977A MS; Agilent, Santa Clara, CA, USA). One microliter of the derivative was injected into the GC operating in splitless mode. The DB-5 ms column with a 10 m DuraGuard capillary (122-5532G; Agilent), which allowed for helium gas to flow at a pressure of 1.1 mL/min, was used to separate the metabolites. The injector temperature was set to 250°C, and column temperature was set to 60°C for 1 min and then increased to 310°C at a rate of 10°C/min. The effluent from the column was automatically inserted into the MS. The detector was operated in electron impact ionization mode (70 eV), and the mass spectrum was recorded after a solvent delay of 6.5 min. The temperature was set to 180°C and 280°C for the quadrupole MS and ion source, respectively.

### 2.6. Quality Control

An internal quality control was performed for all analyses using the following parameters: clear definition of objectives, procedures, norms, and criteria for tolerance limits; corrective actions and recording of activities; and the use of controls to evaluate analytical imprecision [18].

### 2.7. Statistical Analyses

The G^∗^Power version 3.1.7 program was used for sampling the estimates and power of inferences. Initial data filtering was performed using the Microsoft Excel program (Redmond, WA, USA). Statistical normalization and analyses were performed using the MetaboAnalyst program [19]. A fold change calculation and *t*-test were also performed.

For multivariate evaluations, partial least squares-discriminant analysis (PLS-DA) components were determined to discriminate the healthy and periodontitis groups (PLS is a supervised method that uses multivariate regression techniques to extract information for predicting the class of a member (*Y*) using a linear combination of original variables (*X*)). To evaluate the significance of class discrimination, a permutation test was performed. In each permutation, a PLS-DA model was constructed between the data (*X*) and permuted classes (*Y*) using the ideal number of components determined by the model crossvalidation based on the original class.

## 3. Results

### 3.1. Whole Metabolite Profiles

In total, 969 metabolites were identified. Of these, 64 metabolites were detected in at least two of the three replicates and in at least 50% of either group (healthy or periodontitis group); these were considered for further statistical analyses. The metabolite intensities were normalized by self-scaling (Figure S1).

### 3.2. Fold Change Analyses of 64 Commonly Shared Metabolites

Fold change analysis was performed based on the ratio of the mean metabolite intensities in the periodontitis and healthy groups. Those metabolites with an intensity at least 2-fold larger in one group as in the other were considered. We identified nine metabolites with a hazard ratio of >2. Five metabolites were higher in the periodontitis than in the healthy group, with a fold change of >2 (2,3-dihydroxypropyl icosanoate, glycerol, serine, 5-aminovaleric acid, and putrescine), and four were higher in the healthy than in the periodontitis group, with a fold change of <0.5 (lactulose, oxalic acid, 1-benzoyl-2-t-butyl-5-ethyl-3-methyl-5-vinyl-imidazolidin-4-one, and maltose) (Figure 2S and Table 1).

### 3.3. Statistical Significance Assessment

A *t*-test was performed after excluding contaminants, which identified 10 metabolites among the healthy and periodontitis groups (Figure S3 and Table 2). Among these ten metabolites, three were among the five metabolites that showed a fold change > 2 in periodontitis (serine, 5-aminovaleric acid, and putrescine), and two were among the four metabolites that showed a fold change < 0.5 (1-benzoyl-2-t-butyl-5-ethyl-3-methyl-5-vinyl-imidazolidin-4-one and maltose) (Tables 1 and 2).

### 3.4. PLS-DA of Periodontitis and Healthy Groups

**Figure 1:**
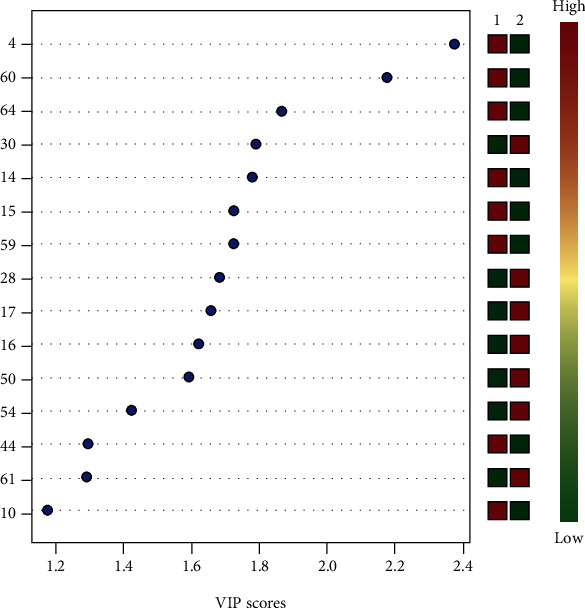
Effect of chronic periodontal disease on the distribution of VIP scores after the metabolome of inflammatory exudate present in the crevicular fluid of old adults patients with periodontal disease. Separation of periodontitis (1-red) and healthy (2-green) groups according to metabolite profile. Numbers 1 and 2 represent the healthy and periodontitis groups, respectively. The concentration of metabolites is represented by the color gradient between green (less concentrated) and red (more concentrated). To the left of the table are the metabolite IDs, identified in Table 3.

According to PLS-DA based on two main components, the periodontitis and healthy individuals were separated based on their metabolic profiles (Figure S4). Furthermore, crossvalidation analysis supported the results and showed that this separation was not random (Figure S5). The metabolites were then ordered based on the variable importance in projection (VIP) index according to their importance in the group separation in PLS-DA (Figure 1 and Table 3).

Overall, 5-aminovaleric acid and serine, which presented a fold change > 2 in the periodontitis group compared to in the healthy group, showed the highest VIPs, whereas the best VIP for a metabolite with a fold change < 0.5 was for 1-monopalmitin.

## 4. Discussion

Periodontal disease is a chronic inflammatory disease that affects the fixation and support structures of teeth. Over the past few decades, great efforts have resulted in advances in the diagnosis and treatment of periodontal disease [20–22]. However, epidemiological studies have shown that this disease remains among the main causes of tooth loss in adults [23], and its progression and development are age-related [24].

Because of improvements in the quality of life, the length of the senescence period, a stage of life in which various biological changes are initiated including in the immune system, has increased. In this study, we identified 969 metabolites correlated with aging in older adults with or without periodontal disease. Of the 969 metabolites, 15 were found to be associated with the presence of periodontal disease, as indicated by a VIP score of >1. Further evaluations showed that two metabolites (5-aminovaleric acid and serine) were found at higher concentrations in the crevicular fluid, which may be useful for predicting the diagnosis of chronic periodontal disease in older adults.

In a recent study, Moeller et al. evaluated longevity in a population and showed that periodontal disease was a mortality factor among the evaluated individuals [25]. Other studies also demonstrated that periodontal disease is associated with several pathological conditions, such as diabetes, cardiovascular diseases, and arthritis [26–28]. Therefore, improved diagnostic methods and new biological markers are needed for periodontal disease; the application and interaction of the -omic approaches will allow us to broaden our perspectives on the molecular mechanisms involved in periodontal disease [29] and enable the optimization of bold diagnostic and prognostic models.

MS with chromatography is useful for predicting periodontal diseases in different progression stages [30]. Considering the high sensitivity of identification and quantification of metabolites in our study, our results support the findings of the previous study.

This is the first study to identify molecules that may predict periodontal disease in older adults. A previous evaluation of younger adults individuals showed that different metabolites are linked with the development of chronic periodontal disease, including ornithine (VIP = 2.57), 5-oxoproline (1.99), valine (1.99), proline (1.35), spermidine (1.15), hydrocinnamate (1.07), histidine (1.04), and cadaverine (1.00) ^11^. In contrast, we showed that in older adult patients with chronic periodontal disease, the following metabolites were more prominent: 5-aminovaleric acid (VIP = 2.37), serine (2.18), 1-monopalmitine, aspartic acid, D-mannitol, putrescine, 1-benzoyl-2-t-butyl-5-ethyl-3-methyl-5-vinyl-imidazolidin-4-one, palmitoleate, maltose, lactic acid, oxalic acid, edetic acid, contaminants, and D-glucose-6-phosphate, of which the first two were remarkably increased. The relationship between periodontitis and increased aminovaleric acid, in addition to lactic acid, certain sugars, and putrescine, a compound associated with tissue decay, has been reported previously [30]. The observed differences in metabolite profiles may be related to the different characteristics of the development of chronic periodontal disease, as well as to the host response to pathogens in the two populations.

In a survey of the literature over a 17-year period (Jan/2000 to Jan/2017), a study identified 90 different components in the crevicular fluid as diagnostic and prognostic markers for periodontal disease, including inflammatory mediators, oxidative stress markers, host-derived enzymes, tissue degradation products, and bone homeostasis mediators [31].

We believe that metabolites from different metabolic pathways can guarantee diagnostic and prognostic specificity and sensitivity. In addition to metabolites from the inflamed site in the periodontium, inflammation-related metabolites from microorganisms are also present, as a marked dysbiosis is established in periodontitis; therefore, these molecules can be biomarkers, pointing at a potential strategy for the prediction, diagnosis, prognosis, and management of personalized periodontal therapy [32].

In our approach, we indicate that the metabolic arrangement found in crevicular fluid can be influenced by senescence, since there is an increased susceptibility to infections and inflammations in old age [33]. In a metabolomic evaluation of crevicular fluid from individuals with a mean age of 39 years (28 to 51 years old), the authors highlighted the association of two components, citramalic acid and N-carbamylglutamate, as markers of chronic periodontitis in an explanatory model with AUC = 87.6% [32]. In another study, metabolomic differences were identified between healthy (26.5 ± 1.7 years old) and periodontitis subjects (mean age = 29.4 ± 4.2 years old), changes in the concentrations of compounds associated with the biosynthesis of amino acids, galactose, and pyrimidine were observed, and correlation between the metabolic profile and microbial community was reported [34].

Multicenter studies or approaches in secondary studies that can guarantee the assessment of strata linked to the pathogen-host relationship, such as immunological status, age, and sociodemographic factors, will certainly provide consistent results in identifying the indicators for the diagnosis and prognosis of periodontal disease.

## 5. Conclusions

Our findings demonstrated that certain metabolites, such as 5-aminovaleric acid, serine, and 3TMS derivative, are likely present in the crevicular fluid of older adults with chronic periodontal disease. Thus, these metabolites can be used as biomarkers for diagnosing periodontal disease in these patients.

## Figures and Tables

**Table 1:** Metabolites with a greater than twofold change in the periodontitis relative to the healthy group.

Metabolite	FC	log_2_ (FC)	HMDB ID
2,3-Dihydroxypropyl icosanoate	20.22	43.381	HMDB11572
Glycerol	6.79	27.643	HMDB00131
Serine	3.93	19.737	HMDB00187
5-Aminovaleric acid	2.54	13.442	HMDB03355
Putrescine	2.17	11.153	HMDB01414
Lactulose	0.48	-10.499	HMDB00740
Oxalic acid	0.47	-10.828	HMDB02329
1-Benzoyl-2-t-butyl-5-ethyl-3-methyl-5-vinyl-imidazolidin-4-one	0.42	-12.655	N/A
Maltose	0.13	-29.432	HMDB00163

FC: fold change; HMDB, Human Metabolome Database; N/A: not available in HMDB.

**Table 2:** Metabolites showing significant differences with a *p* value <0.05 according to *t*-test.

ID	Metabolite	*p* value	HMDB ID
4	5-Aminovaleric acid	0.0008	HMDB03355
60	Serine	0.0024	HMDB00187
30	1-Monopalmitin	0.0137	HMDB31074
14	Aspartic acid	0.0144	HMDB00191
15	D-mannitol	0.0178	HMDB00765
59	Putrescine	0.0179	HMDB01414
28	1-Benzoyl-2-t-butyl-5-ethyl-3-methyl-5-vinyl-imidazolidin-4-one	0.0209	N/A
17	Palmitoleate	0.0230	HMDB03229
16	Maltose	0.0263	HMDB00163
50	Lactic acid	0.0292	HMDB00190

HMDB: Human Metabolome Database; N/A: not available in HMDB.

**Table 3:** Metabolites important for group separation according to partial least squares-discriminant analysis (PLS-DA) and their variable importance in projection (VIP) scores.

ID	Metabolite	VIP score
4	5-Aminovaleric acid	2.37
60	Serine, 3TMS derivative	2.18
64	^∗^Contaminant	1.86
30	1-Monopalmitin	1.79
14	Aspartic acid	1.78
15	D-mannitol	1.72
59	Putrescine	1.72
28	1-Benzoyl-2-t-butyl-5-ethyl-3-methyl-5-vinyl-imidazolidin-4-one	1.68
17	Palmitoleate	1.66
16	Maltose	1.62
50	Lactic acid	1.59
54	Oxalic acid	1.42
44	Edetic acid	1.29
61	^∗^Contaminant	1.29
10	D-glucose-6-phosphate	1.18

^∗^Derivatives of chemical products (example: toothpaste).

## Data Availability

The supplemental data used to support the findings of this study are included within the supplementary information file.

## Supplementary Materials

Supplementary 1 Figure S1. Results of metabolite data normalization by self-scaling. Box and kernel density plots before (left) and after (right) normalization by self-scaling. Boxes indicate the top 50 metabolites, and densities are based on all samples.

Supplementary 2 Figure S2. Results of the fold change (FC) analysis. Metabolites with an intensity at least twice as large (dashed lines indicate the cut offs) in the periodontitis as in the healthy group or vice versa are indicated in pink. Data are presented in logarithmic scale.

Supplementary 3 Figure S3. Results of the metabolite *t*-test. Metabolites with statistical significance (*p* value <0.05) are shown in pink.

Supplementary 4 Figure S4. Separation of periodontitis (1-red) and healthy (2-green) groups according to metabolite profile.

Supplementary 5 Figure S5. Permutation test showing that the metabolic profile separation between the periodontitis and healthy groups was not random (*p* value <0.05).
