# Supplementary figures and images for: Metabolomic Evaluation of Chronic Periodontal Disease in Older Adults
Source: Mediators Inflamm. 2021 Nov 18;2021:1796204. doi: 10.1155/2021/1796204 (PMC8617563; doi:10.1155/2021/1796204)

Before Normalization

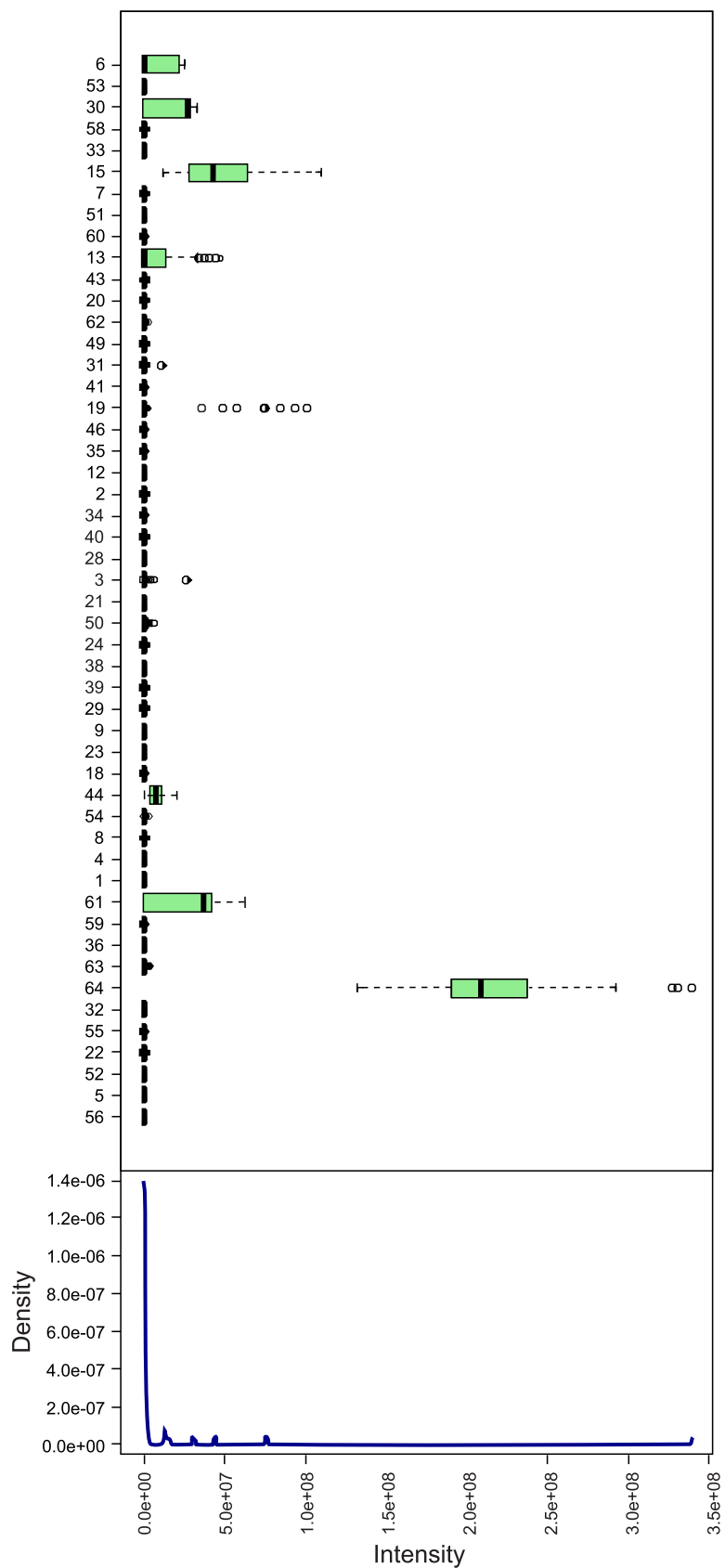

After Normalization

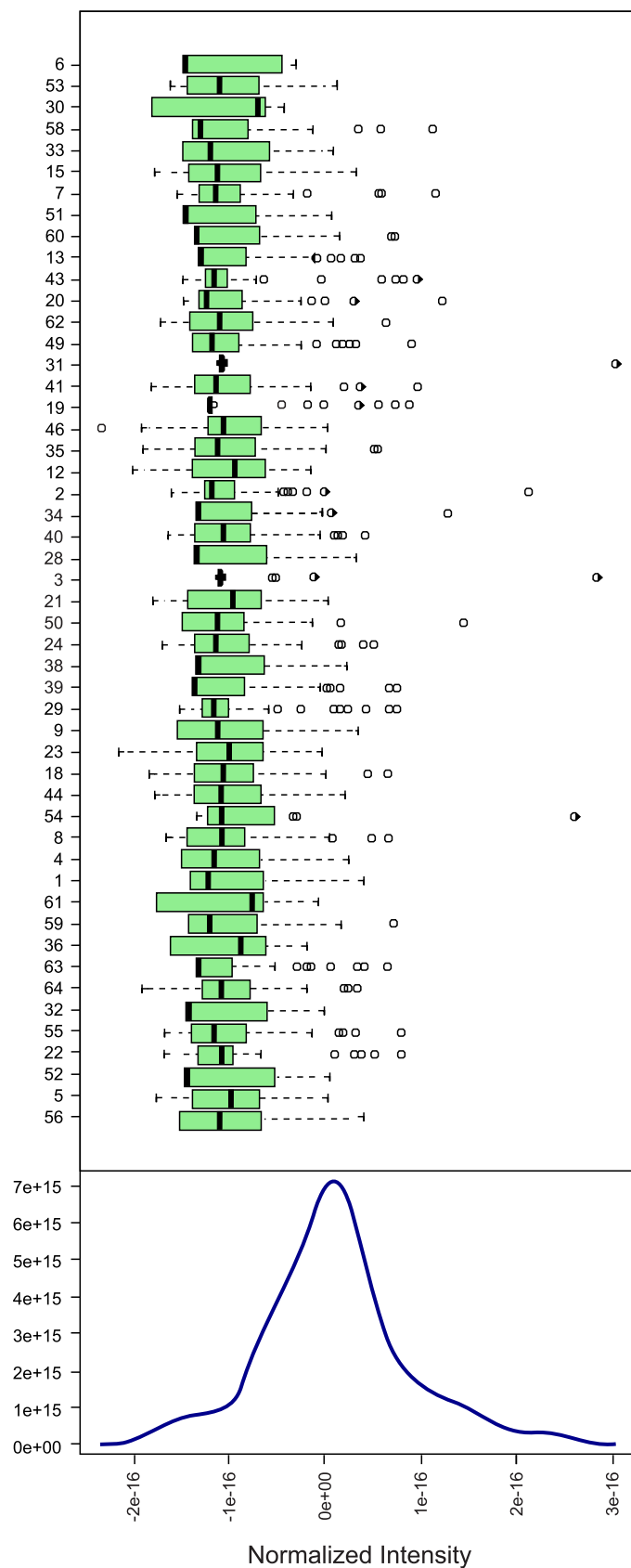

Supplement: Supplementary 1 — Figure S1. Results of metabolite data normalization by self-scaling. Box and kernel density plots before (left) and after (right) normalization by self-scaling. Boxes indicate the top 50 metabolites, and densities are based on all samples. [file 1796204.f1.pdf]

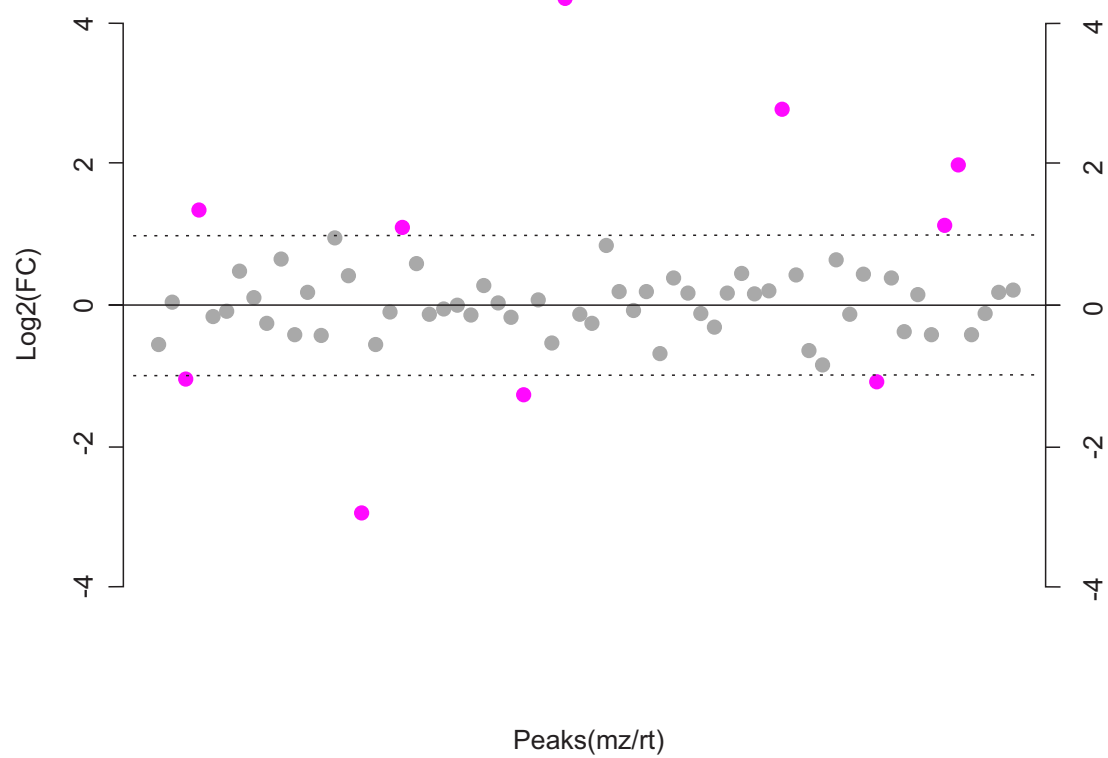

Supplement: Supplementary 2 — Figure S2. Results of the fold change (FC) analysis. Metabolites with an intensity at least twice as large (dashed lines indicate the cut offs) in the periodontitis as in the healthy group or vice versa are indicated in pink. Data are presented in logarithmic scale. [file 1796204.f2.pdf]

# T-Tests

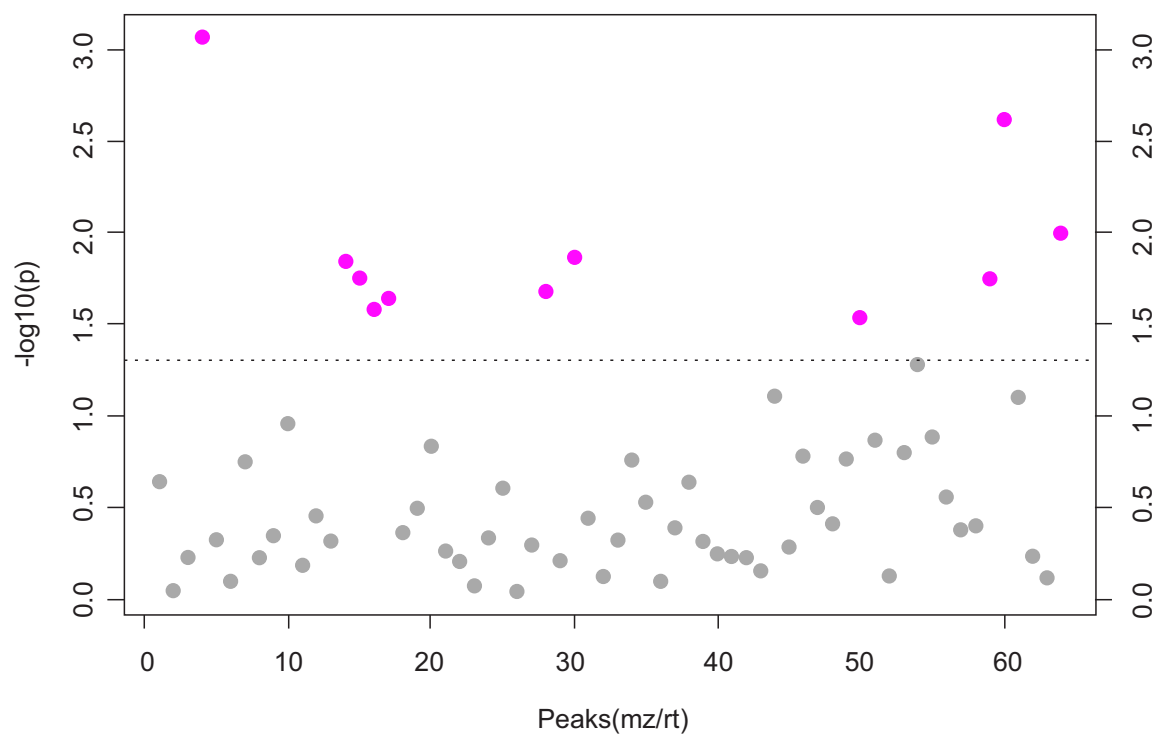

Supplement: Supplementary 3 — Figure S3. Results of the metabolite t-test. Metabolites with statistical significance (p value <0.05) are shown in pink. [file 1796204.f3.pdf]

Scores Plot

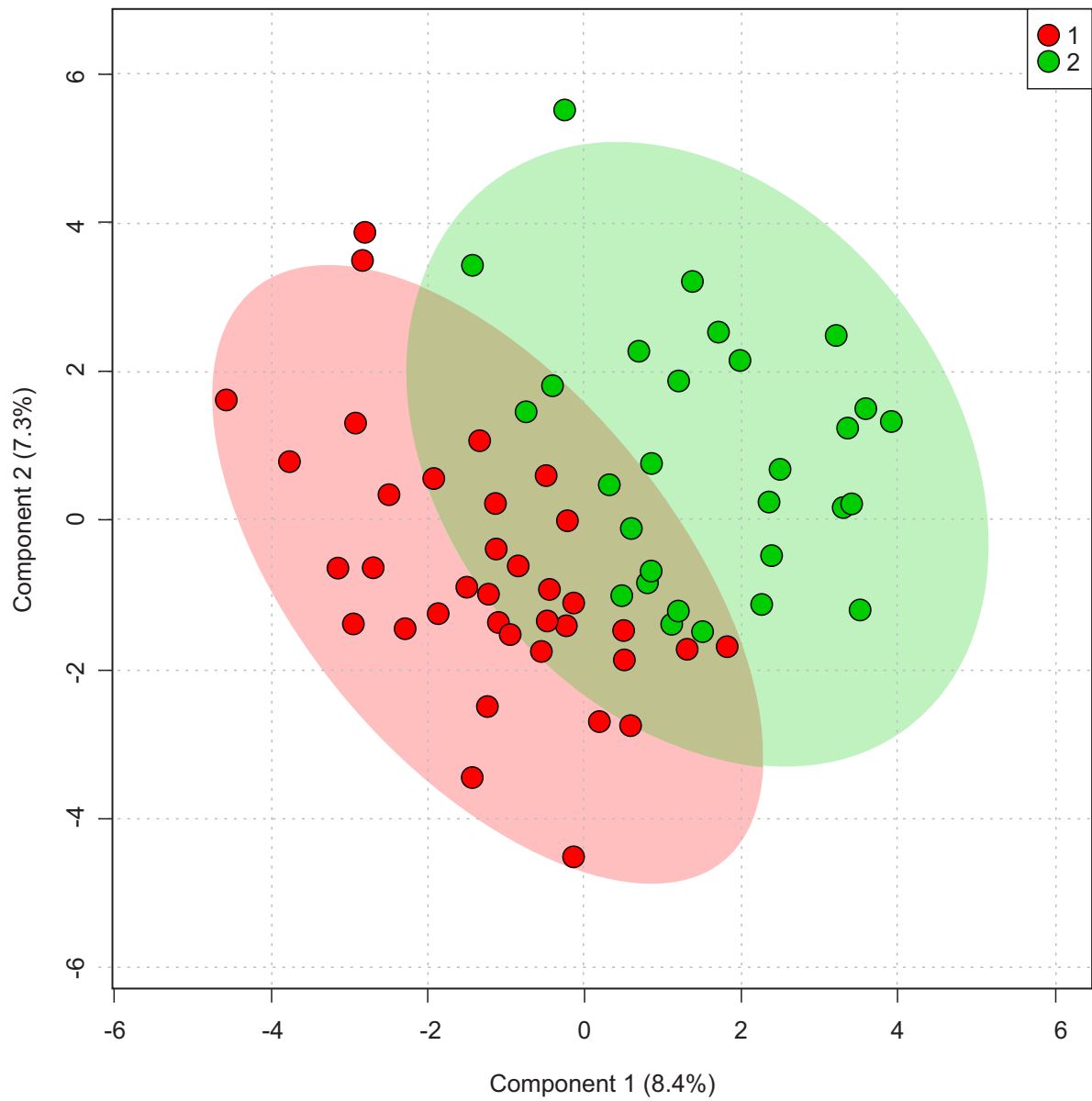

Supplement: Supplementary 4 — Figure S4. Separation of periodontitis (1-red) and healthy (2-green) groups according to metabolite profile. [file 1796204.f4.pdf]

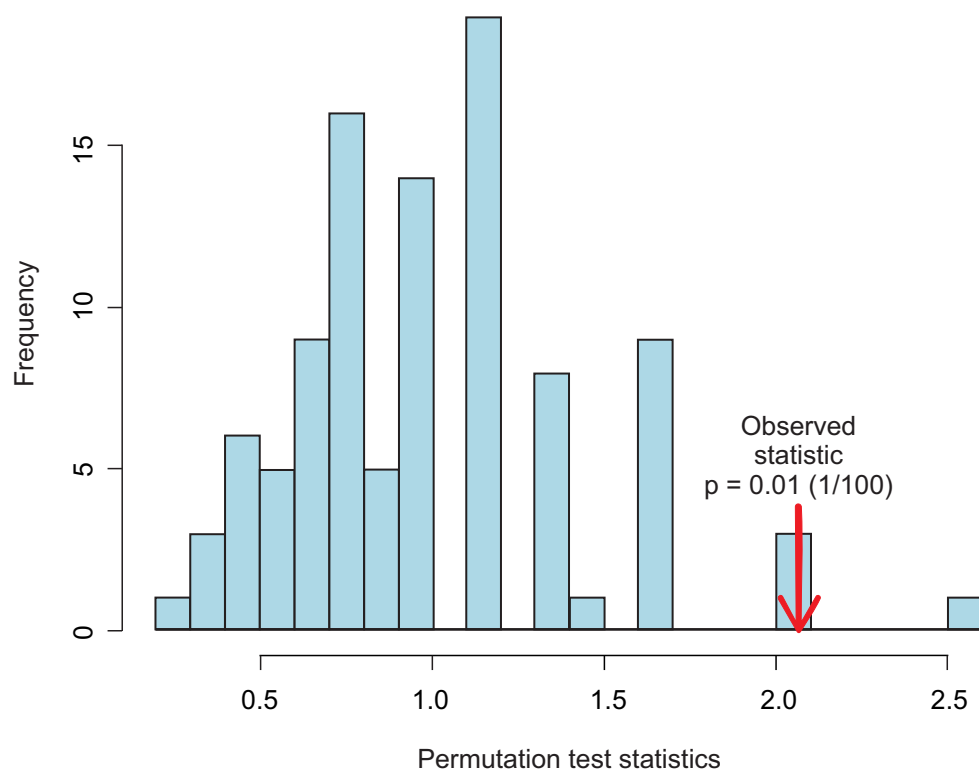

Supplement: Supplementary 5 — Figure S5. Permutation test showing that the metabolic profile separation between the periodontitis and healthy groups was not random (p value <0.05). [file 1796204.f5.pdf]
